# Fibroadenoma Arising in Axillary Ectopic Breast Tissue. A Diagnostic Challenge

**DOI:** 10.7759/cureus.36594

**Published:** 2023-03-23

**Authors:** Nektarios Koufopoulos, Dionysios T Dimas, Ioannis Boutas, Adamantia Kontogeorgi, Kyparissia Sitara, Antigoni Sourla, Christos Kittas, Ioannis L Missitzis

**Affiliations:** 1 Second Department of Pathology, Medical School, National and Kapodistrian University of Athens, Attikon University Hospital, Athens, GRC; 2 Breast Unit, Athens Medical Center, Psychiko Clinic, Athens, GRC; 3 Breast Unit, Rea Maternity Hospital, P. Faliro, Athens, GRC; 4 Third Department of Obstetrics and Gynecology, Medical School, National and Kapodistrian University of Athens, Attikon University Hospital, Athens, GRC; 5 Department of Internal Medicine, “Elpis” General Hospital of Athens, Athens, GRC; 6 Department of Pathology and Laboratory Medicine, Bioiatriki Laboratories, Athens, GRC

**Keywords:** supernumerary breast, accessory breast, ectopic breast tissue, axillary fibroadenoma, fibroadenoma

## Abstract

Ectopic or accessory breast tissue may occur in primitive embryonic milk lines or locations other than the milk line. The same pathology arising in breast tissue may occur less frequently in ectopic breast tissue. Fibroadenomas rarely occur in ectopic breast tissue, with less than 50 reported cases in the English literature, despite being the most common benign breast neoplasms. Diagnosing fibroadenoma in ectopic breast tissue can be challenging due to the lack of clinical suspicion and the atypical findings in imaging studies. Treatment consists of surgical excision. In this manuscript, we present a case of a 24-year-old patient with a fibroadenoma of the left axilla arising in bilateral axillary ectopic breast tissue, and we comprehensively review the literature.

## Introduction

The presence of more than two breasts in human beings with or without a nipple and areola is termed polymastia [1]. Polymastia is synonymous with a supernumerary, accessory, or ectopic breast tissue (EBT). It may occur anywhere along the primitive embryonic milk lines, extending from the axilla to the groin or in locations other than the milk line [2,3]. Females are affected twice as commonly compared to males, with an incidence of 2% to 6% [1,3]. A vast number of pathological changes may occur in EBT, similar to normally located breast tissue albeit with a lower incidence, usually including mastitis, fibrocystic changes, fibroadenoma (FA), phyllodes tumor, intraductal papilloma, and invasive breast carcinoma [2-4]. The latter is the most frequent pathology arising in EBT. It has a poor prognosis due to delayed diagnosis, early lymph node metastasis, and difficulty in surgical management [5]. Fibrocystic disease is the second most common [2,6]. Although FAs are the most common benign breast neoplasms, they rarely occur in EBT, with few reported cases. In this manuscript, we present a case of a 24-year-old woman with a FA arising in EBT in the left axilla, and we perform a comprehensive review of the literature.

## Case presentation

**Figure 1:**
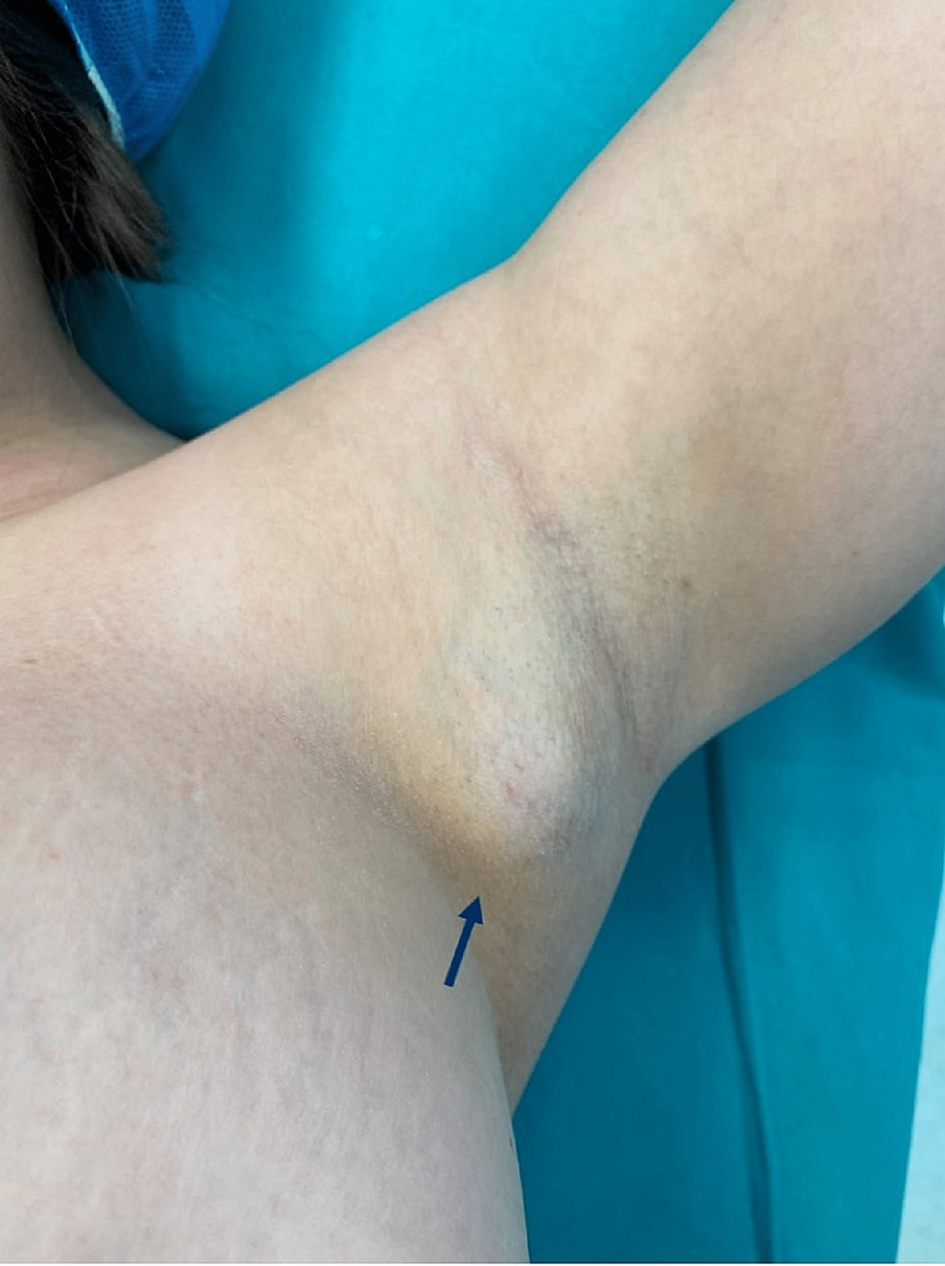
Left axilla with prominent accessory breast tissue and a subcutaneous mobile, firm mass (blue arrow).

A 24-year-old female patient presented to our Breast Unit due to a palpable tumor of her left axilla. On clinical examination, accessory breast tissue was prominent in both axillary regions. A subcutaneous mobile, firm, slightly tender mass was noted in the left axilla, with well-demarcated borders. The lesion was approximately 4 cm in diameter (Figure 1).

Small and elastic lymph nodes were also noted. The rest of the physical examination was normal. The patient did not report a personal or family history of breast disease.

**Figure 2:**
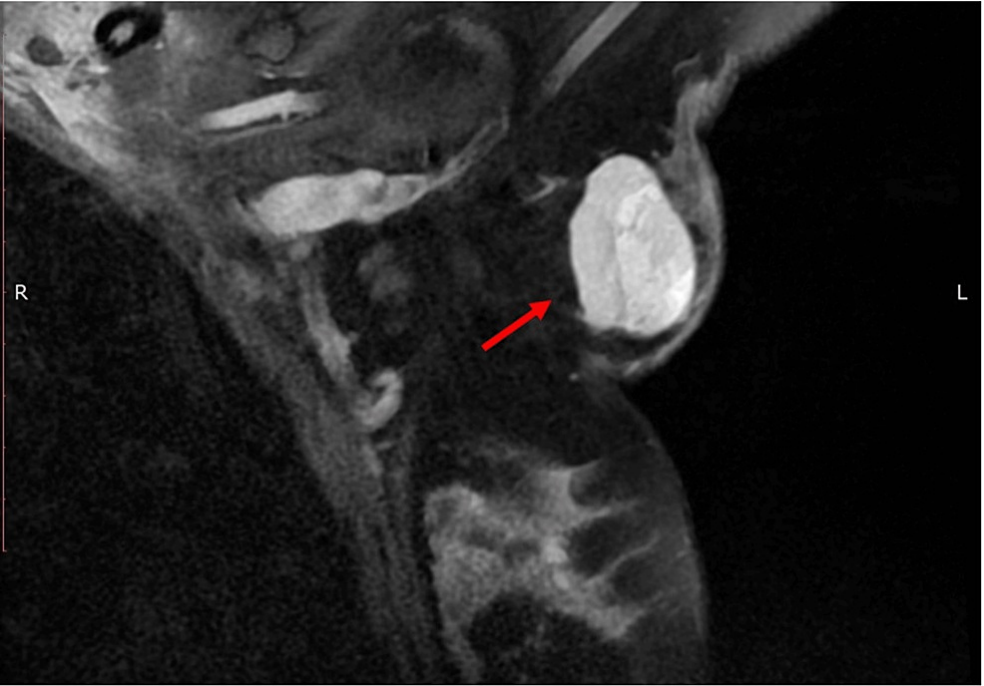
Breast MRI revealed a 4-cm oval mass with microlobulated borders and a type I enhancement curve (red arrow). Non-enhancing internal septations could be seen.

A hypoechoic oval tumor was revealed on ultrasound, measuring 3.77 x 2.11 cm. The tumor was oval, with horizontal orientation and microlobulated margins. Color Doppler detected blood flow within small vessels. Breast MRI demonstrated a strongly but heterogeneously enhancing, microlobulated, oval mass with a maximum diameter of 4 cm and a type I enhancement curve. Non-enhancing internal septations could be seen (Figure 2).

**Figure 3:**
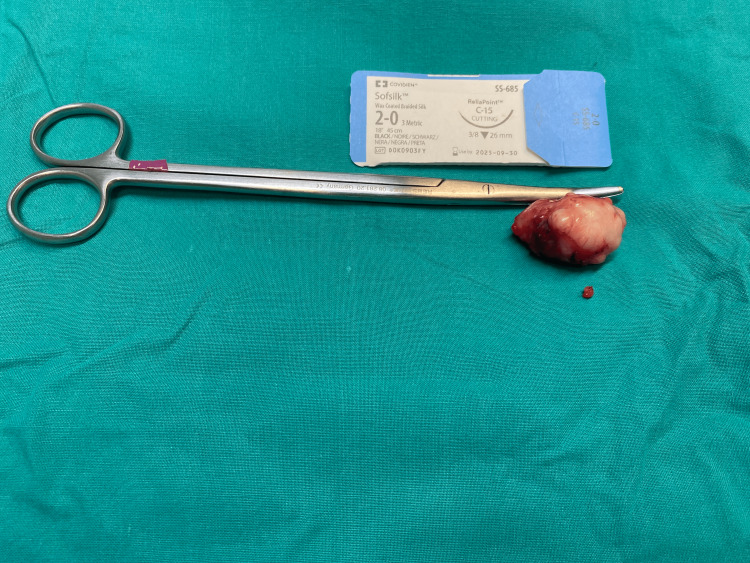
Macroscopic photograph of the surgical specimen

Based on these characteristics, the differential diagnosis included ectopic breast fibroadenoma or phyllodes tumor, subcutaneous soft tissue sarcoma, or even benign peripheral nerve tumor (schwannoma). A fine-needle aspiration biopsy (FNAB) was performed, and cytologically, the mass was diagnosed as a proliferative breast lesion without atypia. Subsequently, surgical tumor excision was performed under general anesthesia (Figure 3).

**Figure 4:**
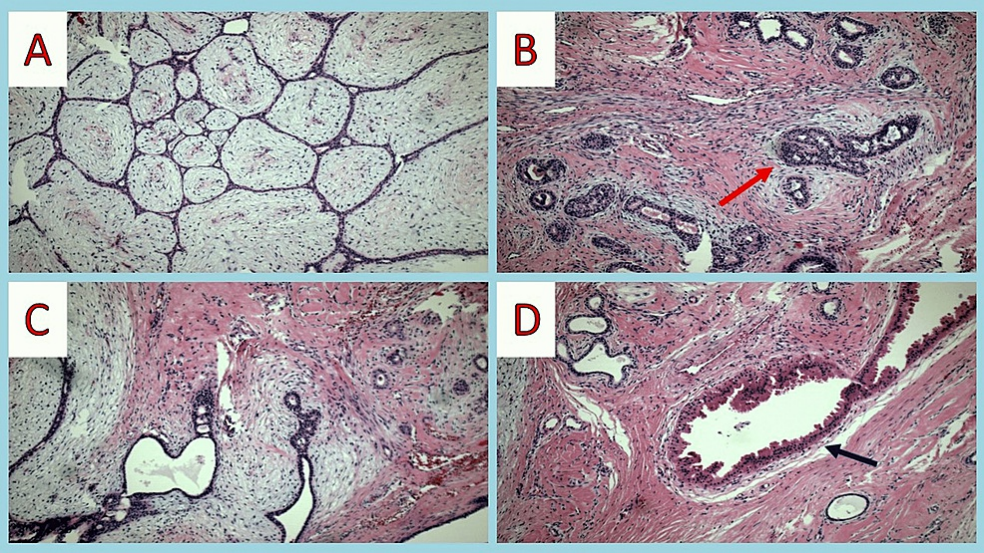
(A) Intracanalicular pattern-stromal growth around ducts – expansion of the stroma compresses the ductal element with the formation of slit-like spaces. (B-D) A few patent ducts show epithelial hyperplasia (red arrow) and apocrine metaplasia of ductal epithelium (blue arrow).

On gross examination, the tumor was encapsulated, well-circumscribed, lobulated, pink/white, and of soft elastic consistency with a maximum diameter of 4 cm. Microscopic examination revealed a biphasic neoplasm consisting of epithelial duct structures lined by cuboidal epithelial luminal cells without atypia surrounded by a layer of myoepithelial cells. A few patent ducts show epithelial hyperplasia and apocrine metaplasia of ductal epithelium. Expansion of the stroma resulting in compression of the ductal element with the formation of slit-like spaces was also observed. Stromal cells lacked significant cellularity or atypia, and mitoses were not identified (Figures 4A-4D).

Based on these findings, our diagnosis was biphasic fibroepithelial neoplasm consistent with FA (intracanalicular pattern) arising in EBT. Follow-up examination of the patient three years postoperatively is without findings.

## Discussion

The axilla is the most frequently reported EBT location, accounting for 60%-70% of all cases [6]. Other anatomical areas reported include the vulva, perineum, face, posterior neck, thigh, shoulder, and foot [2,7,8]. EBT occurs sporadically, but hereditary predisposition has been reported [2]. Concerning the embryogenesis of EBT, there are two theories. The first claims a lack of involution of the embryologic mammary ridges and displacement of the milk line, while the second supports that it results from modified apocrine sweat glands [9]. Kajava published a classification system for EBT in 1915 that remains in use (Table 1).

**Table 1:** Kajava classification

Class I	Complete breast, including glandular tissue, nipple, and areola.
Class II	Breast tissue and nipple, without areola.
Class III	Breast tissue and areola, without a nipple.
Class IV	Breast tissue, without nipple or areola.
Class V	Nipple and areola, without breast tissue.
Class VI	Nipple, without breast tissue or areola (Polythelia).
Class VII	Areola, without breast tissue or nipple (Polythelia areolaris).
Class VIII	Hair without breast tissue, nipple, or areola (Polythelia pilosa).

Clinically, in most cases, the presence of EBT manifests as a palpable lump [2]. It is associated with cyclic changes during menstruation and usually appears in pregnancy [10,11]. The correct diagnosis of EBT is essential because, in particular, polythelia can be associated with anomalies of the urinary tract, such as failure of kidney formation, supernumerary kidneys, hydronephrosis, polycystic kidney ureteric stenosis, duplicate renal arteries, and renal carcinoma or, less commonly, the cardiovascular system [1,2,5].

Even though FA is a common cause of breast lumps in young women, with a peak incidence between 20 and 30 years [12], it rarely occurs in EBT. We extensively searched PubMed, PubMed Central, and Google Scholar until January 15, 2023. Our search yielded 48 manuscripts describing 49 cases after excluding publications in languages other than English [1-3,5-7,9-51].

Regarding the demographic data, patient age ranged from 10 to 58 years (mean 26.4, median 28), while tumor size ranged from 1 to 11 cm (mean 3, median 3). Thirty cases occurred in the right axilla, seventeen in the left, and four were bilateral. In some cases, one or more concurrent breast FAs were noted [6,7,16,46]. Two patients had a previous history of renal transplantation and were receiving Cyclosporine A [14,16]. Lages et al. reported a case of axillary FA in a patient with macroprolactinoma [17]. In a few instances, other pathologies, such as duct ectasia, fibrocystic disease, and apocrine metaplasia, were found simultaneously with FAs in the same axilla [22,43]. Lopez et al. reported a case in which the patient developed two vulvar FAs four years after the diagnosis of axillary FA [43].

Patients with axillary FAs usually seek medical attention after a history of a palpable lump that ranges from a few days to several years. In some cases, they report symptoms of pain, tenderness, and discomfort [23-25,47]. On physical examination, fibroadenomas are firm in consistency, well-defined, tender or non-tender, and freely mobile. A summary of the clinical characteristics of axillary fibroadenomas can be seen in Table 2.

**Table 2:** Clinical characteristics of axillary fibroadenomas * age mentioned in the manuscript as in 20s

Case nr.	Authors	Year	Age	Tumor size	Laterality
1	Aughsteen et al. [5]	2000	28	3.0 cm	Right
2	Conde et al. [13]	2004	39	1.2 cm	Right
3	Coras et al. [3]	2005	23	2.0 cm	Right
4	Ciralik et al. [1]	2006	23	4.0 cm	Right
5	Yarak et al. [14]	2007	33	3.0 cm	Right
6	Odike et al. [15]	2008	34	1.5 cm	Right
7	Sawa et al. [6]	2010	41	3.8 cm	Right
8	Gentile et al. [12]	2010	58	6.5-6.0 cm	Bilateral
9	Mukhopadhyay et al. [7]	2010	17	4.0 cm	Left
10	Darwish et al. [16]	2010	35	3.0 cm	Right
10	Lages et al. [17]	2011	30	2.6 cm	Left
11	Rizvi et al. [9]	2012	32	NA	Left
12	Val-Bernal et al. [18]	2012	29	2.8 cm	Right
13	Borsook et al. [19]	2013	10	7.5 cm	Left
14	Amaranathan et al. [2]	2013	31	2.0 cm	Right
15	Goyal et al. [20]	2014	23	1.0 cm	Left
16	Rajkumar et al. [21]	2014	33	3.0-3.0	Bilateral
17	Singh et al. [22]	2015	30	6.0 cm	Right
18	Paziar et al. [10]	2015	28	5.0 cm	Left
19	Vidyasagar et al. [23]	2015	24	3.0 cm	Left
20	Jethwani et al. [24]	2015	25	4.0 cm	Right
21	Kohli et al. [25]	2015	34	2.8 cm	Right
22	Seo et al. [26]	2015	20	8.5 cm	Right
23	Tiwary et al. [27]	2015	18	3.0 cm	Left
24	Tiwary et al. [27]	2015	21	NA	Right
25	Surd et al. [11]	2016	17	3.0 cm	Right
26	Tamaknand et al. [28]	2016	21	3.0 cm	Left
27	Arora [29]	2017	38	11.0 cm	Right
28	Yilmaza et al. [30]	2017	13	4.0 cm	Right
29	Rani and Mehrolia [31]	2017	25	1.0 cm	Left
30	Gajaria and Maheshwari [32]	2017	37	2.0 cm	Right
31	Korumilli et al. [33]	2018	48	2.0 cm	Right
32	Amir et al. [34]	2018	34	3.0 cm	Right
33	Nigam et al. [35]	2018	21	3.0 cm	Right
34	Sanjay and Ujjwal [36]	2018	26	4.5 cm	Left
35	Srinivasan et al. [37]	2018	38	5.0 cm	Right
36	Trisal et al. [38]	2018	19	4.0 cm	Left
37	Motsumi et al. [39]	2018	30	4.0 cm	Right
38	Erbaba et al. [40]	2019	41	6.0/4.0 cm	Bilateral
39	Ravikanth et al. [41]	2020	42	1.2 cm	Left
40	Krishna et al. [42]	2020	28	3.5/3,5 cm	Bilateral
41	Lopez et al. [43]	2020	29	2.5 cm	Right
42	Laporte et al. [44]	2020	23	2.0 cm	Right
43	Kurt et al. [45]	2021	27	2.5 cm	Left
44	Pshtiwan et al. [46]	2022	38	1.2 cm	Right
45	Yefter and Shibiru [47]	2022	28	5.0 cm	Left
46	Tee et al. [48]	2022	*	3.0 cm	Right
47	Fadhil et al. [49]	2022	19	5.7 cm	Right
48	Hota and Kumari [50]	2022	42	2.0 cm	Left
49	Virji et al. [51]	2022	37	2.0 cm	Left
50	Our case	2023	24	4.0 cm	Left

The occurrence of an axillary mass can be diagnostically challenging. The differential diagnosis includes infectious lesions, benign tumors, and primary or metastatic malignancies. Infectious lesions may include reactive lymphadenitis or lymphadenopathy due to a local infection, tuberculosis, cat-scratch disease, and suppurative hidradenitis. Benign tumors include lipomas, sebaceous cysts, and vascular lesions. Malignancies include breast carcinoma or melanoma metastasis to axillary lymph nodes, lymphoma, rhabdomyosarcoma, squamous cell carcinoma, and neuroendocrine tumors [3,18].

Imaging studies may be helpful, but occasionally they may show atypical findings with suspicious characteristics such as irregular shape, lack of circumscription, and posterior shadowing suggesting a malignant tumor [52]. Histopathological examination is important for the correct diagnosis since most cases are clinically misdiagnosed due to a lack of suspicion. It is unsurprising that, in some cases, the clinical diagnosis was a malignant tumor [6,47].

FAs arising in EBT are histologically identical to their counterparts in the breast. The case reported by Val-Bernal et al. showed hypocellular sclerotic fibroma-like areas [18]. One case concerned a juvenile FA, while several described cases must be classified as giant FAs due to large size (> 5cm).

The recommended treatment for these cases is complete surgical excision, not only for cosmetic and functional reasons but also because invasive carcinoma may occur in EBT [30].

## Conclusions

In summary, we have presented a case of FA arising in the axilla, and we have reviewed the literature extensively. It is important to remember that in any lump appearing in the milk line, the presence of FA rising in EBT must be considered in the differential diagnosis. The diagnostic methods used must be similar to FAs arising in the breast. The treatment should be surgical excision.
